# The construction and analysis of ceRNA network and patterns of immune infiltration in lung adenocarcinoma

**DOI:** 10.1186/s12885-021-08932-z

**Published:** 2021-11-16

**Authors:** Jinglong Li, Wenyao Liu, Xiaocheng Dong, Yunfeng Dai, Shaosen Chen, Enliang Zhao, Yunlong Liu, Hongguang Bao

**Affiliations:** 1 Department of Thoracic Surgery, The Second Affiliated Hospital of Qiqihar Medical College, No.37, West Zhonghua Road, Jianhua District, Qiqihar, 161000 Heilongjiang Province China; 2 Laboratory Department of the Second Affiliated Hospital of Qiqihar Medical College, Qiqihar, Heilongjiang Province China

**Keywords:** Competitive endogenous RNA (ceRNA), Lung adenocarcinoma (LUAD), MicroRNA, Risk-assessment model, Tumor-infiltrating immune cells (TIICs)

## Abstract

**Background:**

Competitive Endogenous RNA (ceRNA) may be closely associated with tumor progression. However, studies on ceRNAs and immune cells in LUAD are scarce.

**Method:**

The profiles of gene expression and clinical data of LUAD patients were extracted from the TCGA database. Bioinformatics methods were used to evaluate differentially-expressed genes (DEGs) and to form a ceRNA network. Preliminary verification of clinical specimens was utilized to detect the expressions of key biomarkers at the tissues. Cox and Lasso regressions were used to identify key genes, and prognosis prediction nomograms were formed. The mRNA levels of 9 genes in the risk score model in independent clinical LUAD samples were detected by qRT-PCR. The interconnection between the risk of cancer and immune cells was evaluated using the CIBERSORT algorithm, while the conformation of notable tumor-infiltrating immune cells (TIICs) in the LUAD tissues of the high and low risk groups was assessed using the RNA transcript subgroup in order to identify tissue types. Finally, co-expression study was used to examine the interconnection between the key genes in the ceRNA networks and the immune cells.

**Result:**

A ceRNA network of 115 RNAs was established, and nine key genes were identified to construct a Cox proportional-hazard model and create a prognostic nomogram. This risk-assessment model might serve as an independent factor to forecast the prognosis of LUAD, and it was consistent with the preliminary verification of clinical specimens. Survival analysis of clinical samples further validated the potential value of high risk groups in predicting LUAD prognosis. Five immune cells were identified with significant differences in the LUAD tissues of the high and low risk groups. Besides, two pairs of biomarkers associated with the growth of LUAD were found, i.e., E2F7 and macrophage M1 (*R* = 0.419, *p* = 1.4e^− 08^) and DBF4 and macrophage M1 (*R* = 0.282, *p* < 2.2 e^− 16^).

**Conclusion:**

This study identified several important ceRNAs, i.e. (E2F7 and BNF4) and TIICs (macrophage M1), which might be related to the development and prognosis of LUAD. The established risk-assessment model might be a potential tool in predicting LUAD of prognosis.

**Supplementary Information:**

The online version contains supplementary material available at 10.1186/s12885-021-08932-z.

## Introduction

Lung cancer (carcinoma) is one of the most prevalent tumors with rapid progression ability, high metastatic potential, high morbidity, and fatality [1]. The World Health Organization reported in 2018 that incidences and death rates of lung cancer were 11.6 and 18.4% respectively. There are two main subtypes of lung carcinoma, namely, small-cell lung cancer (SCLC) and non-small-cell lung cancer (NSCLC). Pathologically, there are several subtypes of NSCLC, namely, lung adenocarcinoma (LUAD), squamous carcinoma (LUSD), and adenosquamous carcinoma (AC). Among these NSCLCs, LUAD is the most prevalent subtype in histology. Its rapid progression ability mainly due to the high metastatic nature of the tumor. This is the most common reason for treatment to fail [2]. LUAD is one of the most critical malignancies, which is often detected at the progressive stage with a poor clinical prognosis [3]. Numerous genetic and molecular biomarkers, such as protein-coding and non-coding genes are often utilized as diagnostic and remedial means for LUAD [4–8]. Among these biomarkers, the competitive endogenous RNAs (ceRNAs) and tumor-infiltrating immune cells (TIICs) are potentially the most important ones, as they could affect tumor development. However, studies on the ceRNAs are scarce with poor clinical prognosis, and the factors related to the LUAD progression remain largely unknown.

The ceRNA network assumes that RNA transcripts containing microRNA (miRNA) response elements could seal miRNA from other targets obtaining the same miRNA response elements, thus, modulating its expression and life processes [9]. Earlier studies found that the ceRNA networks were involved in the development, metastasis, and prognosis of lung cancers, such as linc00665 and miR-98 [10] networks. Besides, the presence of TIICs, especially the leukocytes, supervise the immune system activities, may played a marjor role in the development, progression, and metastasis of neoplastic cells, including LUAD [11–13]. However, only a handful of investigations focused on the regulatory mechanism between the ceRNA network and TIICs, but not LUAD [14].

This study aimed to construct a ceRNA network risk assessment model that could be used for prognosis prediction and immune infiltration analysis of LUAD. A LUAD-related ceRNA network was established based on gene expression profiles to identify key LUAD genes, and the prognostic values of these genes were determined using a Cox proportional-hazard model. Also, this study estimated the proportion of TIICs in LUAD tissues in the high and low risk groups using the Cell Type Identification Estimation of Relative Subpopulation RNA Transcription (CIBERSORT) algorithm. Moreover, co-expression analysis was used to forecast the occurrence and growth of LUAD. The mechanism of LUAD development was also considered. The risk-assessment model and the established mechanism of regulation might provide us with a potential insight for predicting the clinical occurrence and development of LUAD.

## Materials and methods

### Source of data and analysis of disparate gene expression

In this study, data were extracted from The Cancer Genome Atlas (TCGA; https://gdc-portal.nci.nih.gov/). These data comprised of the clinical information of LUAD patients and the profiles of long non-coding RNA (lncRNA), messenger RNA (mRNA), and microRNA (miRNA). Data were organised by different expression profiles, ID conversion, filtering, merging, correction, and clinical information. Demography and other information of patients, survival endpoints, and the histology and tumor stage of the LUAD were also acquired. Genes that showed no expression in LUAD (genes that did not show in tests and control groups) were seperated. Differentially expressed RNAs, including Differentially Expressed LncRNAs (DELs), Differentially Expressed miRNAs (DEMs) and Differentially Expressed mRNAs (DEGs) were determined utilizing the edgeR method. The minimum and maximum regulated genes, | log 2 fold change (log_2_FC) | > 1.0, and the false discovery rate (FDR) < 0.05 were considered as thresholds.

### Patient selection

Patients who underwent surgical resection of primary LUAD at The Second Affiliated Hospital of Qiqihar Medical College, Qiqihar, Heilongjiang Province, China between Oct. 2019 and Aug. 2021 were selected for this study. Tumor and non-tumor specimens (located > 2 cm from the tumor margin) obtained from the enrolled patients were analyzed by RT-qPCR for nine genes. All patients did not receive chemotherapy or radiotherapy prior to surgery. Pathological diagnosis was made by 2 pathologists. The Second Affiliated Hospital of Qiqihar Medical College institutional review board approved the study protocol, and all patients provided written informed consent.

### Construction of LUAD-associated ceRNA networks and identification of genes associated with prognosis

Before the analysis of statistics, experimentally validated information on miRNA-mRNA interactions based on miRTarBase was downloaded from the experimental modules of the database Lncbase v.2, along with the lncRNA-miRNA interaction data. All interactions were validated by direct molecular mechanisms, such as luciferase reporter experiments and immunoprecipitation. To simultaneously regulate lncRNAs and mRNAs, the hypergeometric testing and correlation analysis of miRNAs was performed to obtain DEM-DEG pairs. Axes of DEM-DEG were obtained to build the ceRNA network and visualised using the software Cytoscape (version 3.6.1; www.cytoscape.org/). The two-sided univariate Cox regression was analysed using the software survival package in R (version 3.5.1, Institute for Statistics and Mathematics, Vienna, Austria; www.r-project.org) at the significance level 0.05 to identify the overall survival (OS) and disease-free survival (DFS) related to DEMs and DEGs.

In order to test the modeling derived from different genes, the key genes were screened with Cox and Lasso regressions to create a predictive point chart. The Cox model encompassed all elements of the ceRNA network, and the Lasso regression was used to find the overfitting of the model. The values of all biomarkers were determined using the Cox proportional-hazard model. A histogram was created with the model to forecast the survivalbility of LUAD patients. Calibration curves and receiver operating characteristic curves (ROCs) were used to assess the precision and discriminatory power of the chart. Finally, each biomarker was validated using the Kaplan-Meier survival analysis method.

### Immune risk assessment of key RNAs, CIBERSORT estimation, and the validation of initial clinical specimens

Univariate and multivariate Cox regressions were used. Factors like age, sex, tumor stage, TNM, and risk score were performed to examine if the constructed immune risk model was unconstrained by clinicopathological parameters. The CIBERSORT algorithm (citation) was used to identify cell types by estimating the relative subset of RNA transcripts of 22 cell types to determine the relationship between risk and immune cells in LUAD. Cell types in samples with a CIBERSORT output of *p* < 0.05 were further identified by determining the relative subset of RNA transcripts. The Wilcoxon rank-sum test was employed to examine whether the proportion between high and low risk groups in LUAD tissue was different by identifying the immune-infiltrating cells. Expressions of the key genes in the tissues were verified through searching over the database of The Human Protein Atlas (https://www.proteinatlas.org/). The immunohistochemical results of key genes in ceRNA were compared.

### RNA extraction and qRT-PCR

Total RNA was extracted from LUAD tissues with Trizol Reagent (Invitrogen, Carlsbad, CA, USA), which was reversely transcribed with RT reagent Kit gDNA Eraser (TaKaRa). And then, cDNA expression levels were detected by SYBR-Green (TaKaRa) and RT-qPCR analysis with GAPDH as internal reference. The primers were shown in Table 1. PCR amplification was carried out in a formula of three Wells. All experiments were repeated three times and genes’ relative expression levels were studied with 2^-△△Ct^.

**Table 1:** RT-qPCR primers

Gene	Primers
DBF4	Forward (F): 5′-GTCTCCGCAGACTCCAAAGT-3’
	Reverse(R): 5′-CCGTTTCTTTCTTCACCGGC-3’
CPS1	Forward (F): 5′-AGCCGAGGCCCATGCCACAA-3’
	Reverse(R): 5′-AGGAGCCTGATGCCAGGTCTTGA-3’
CDC14A	Forward (F): 5′-CCGACCCTCCTACACCGGGCT-3’
	Reverse(R): 5′-AGGAGAGCAGGGGGCTTCCA-3’
CCT6A	Forward (F): 5′-GGGGCCCAAGGGCACCATGAAG-3’
	Reverse(R): 5′-AGGATGAAGGCCCATTTCGTGAAGC-3’
SLC16A1	Forward (F): 5′-AGCCGGACCCTGGGCCCCGTGGAA-3’
	Reverse(R): 5′-CGCGCCGCGTGCCGCCGGCTGTTA-3’
E2F7	Forward (F): 5′-GGTGGAATTGAAGCTGCTGCGCTA-3’
	Reverse(R): 5′-CAGTGTAGGGCACACACAGCCTCT-3’
GPR37	Forward (F): 5′-CGGCAGGGACGCCTGGGGACCGGGA-3’
	Reverse(R): 5′-CGGCTGCCGACGCCTCCGCCCCTCT-3’
SNHG3	Forward (F): 5′-TGTGGAGGTGGCTGTGGTGACATC-3’
	Reverse(R): 5′-ACCCAAGGGGGACCCACCTGAGAC-3’
hsa-miR-326	Forward (F): 5′-TGGGCTGGAGGCAGGGCCTTTGT-3’
	Reverse(R): 5′-CGGGGCTGGAGGAAGGGCCCAGA-3’
GAPDH	Forward (F): 5′-TGTGTCCGTCGTGGATCTGA-3’
	Reverse(R): 5′-CCTGCTTCACCACCTTCTTGA-3’

### Statistical analyses

The software R was used to perform all statistical analyses, such as preprocessCore package, edgeR package, rms package, and ggplot2 package. The survival analysis was performed using the Fisher’s precision test for the high and low risk score models. Only a two-sided *p* value < 0.05 was considered statistically significant.

## Result

### Identifying genes with markedly different expression

**Fig. 1:**
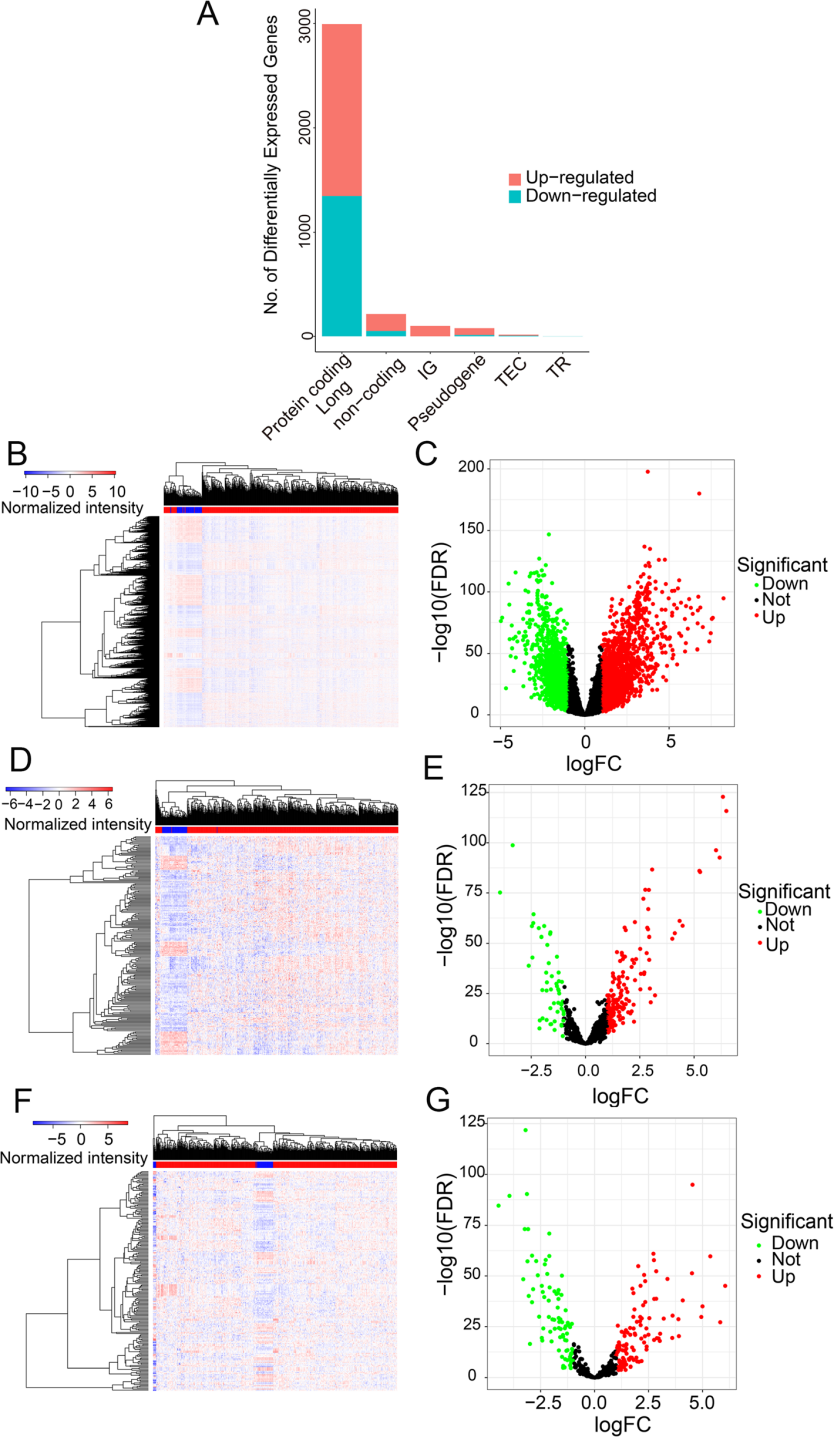
Identification of differentially expressed genes (DEGs) between LUAD and adjacent normal tissue A total of 13,709 lncRNAs, 1479 miRNAs and 19,413 mRNAs from the TCGA database were identified 3406 of which were differentially expressed genes (A), including 2994 protein-coding genes (B, C), 198 miRNAs (D, E) and 214 lncRNAs (F, G). The cutoffs were |log_2_FC| > 1.0 and FDR < 0.05.

A total of 694 samples, including 535 LUADs and 59 adjacent normal tissues were downloaded from TCGA, with a total of 19,413 mRNAs, 13,709 lncRNAs, and 1479 miRNAs were identified from LUADs and paraneoplastic tissues (Fig. 1A). Using |log_2_FC| > 1.0 and FDR < 0.05 as threshold value, variations were detected in 2994 DEGs (1649 up- and 1345 down-regulated; Fig. 1B and C), 198 expressions of individual miRNAs (111 up- and 87 down-regulated; Fig. 1D and E), and 214 lncRNAs (163 up- and 51 down-regulated; Fig. 1F and G).

### Construction of the ceRNA network and identification of genes associated with prognosis

**Fig. 2:**
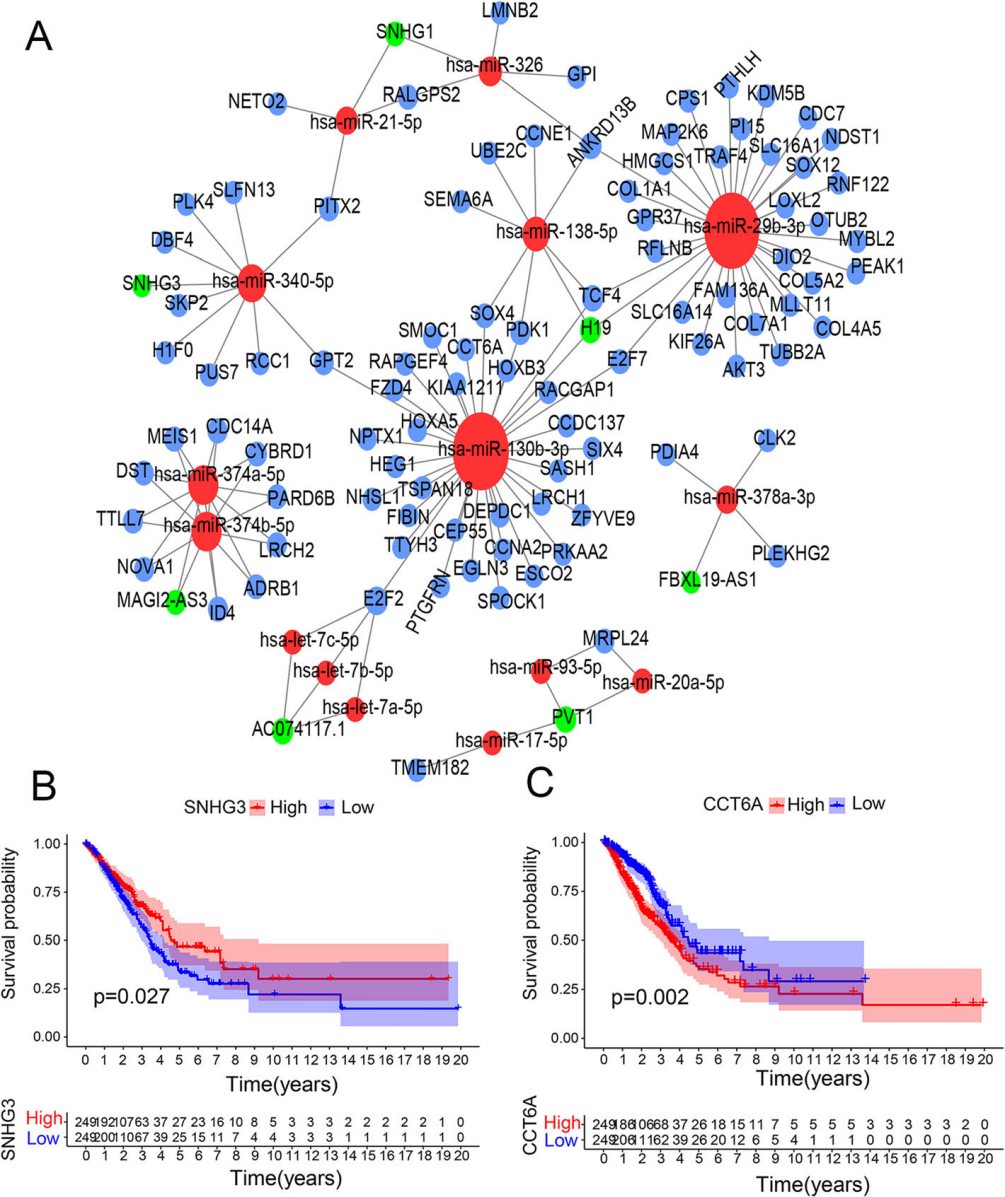
Construction of LUAD-associated ceRNA network and survival curves of principal genes in the ceRNA network Construction of LUAD-associated ceRNA network, (B-C) Kaplan Meier survival curves of principal genes (SNHG3, CCT6A) in the ceRNA network.

The ceRNA network was constructed in this study with 115 genes (seven lncRNAs, 15 miRNAs, and 93 mRNAs) in order to examine the pattern of differential gene regulation (Fig. 2A). Table 2 shows the ceRNA network with a hypergeometric *p -*value < 0.05 as the cut-off value. In addtion, the results of gene survival analysis were screened for prognostic significance of ceRNA nodes with *p* < 0.05 as the cut-off value. As a result, this study demonstrated the survival curve of two genes, SNHG3 and CCT6A, respectively (Fig. 2B-C). Both curves show that the expression levels of SNHG3 and CCT6A were associated with the overall survivalbility of patients with LUAD.

**Table 2:** Results of hypergeometric test and correlation analysis of ceRNAs network

lncRNAs	Genes	miRNAs	Correlation p	Hypergeometric test p
PVT1	TMEM182	hsa-miR-17-5p	6.81E-05	0.049992152
PVT1	MRPL24	hsa-miR-20a-5p, hsa-miR-93-5p	1.12E-19	0.010852636
SNHG1	PITX2	hsa-miR-377-3p, hsa-miR-21-5p	2.21E-05	0.010361047
SNHG1	RALGPS2	hsa-miR-326, hsa-miR-377-3p, hsa-miR-21-5p	0.017396142	0.004835561
SNHG1	GPI	hsa-miR-326, hsa-miR-330-5p	1.09E-05	0.001165794
SNHG1	LMNB2	hsa-miR-326, hsa-miR-330-5p	1.13E-08	0.002308944
SNHG1	NETO2	hsa-miR-377-3p, hsa-miR-21-5p	7.18E-05	0.041029046
SNHG1	ANKRD13B	hsa-miR-326, hsa-miR-330-5p	2.48E-08	0.041029046
MAGI2-AS3	ADRB1	hsa-miR-374b-5p, hsa-miR-374a-5p	8.29E-10	0.033354261
MAGI2-AS3	CYBRD1	hsa-miR-374b-5p, hsa-miR-374a-5p	2.17E-49	0.012975462
MAGI2-AS3	CDC14A	hsa-miR-374b-5p, hsa-miR-374a-5p	2.96E-19	0.006618532
MAGI2-AS3	ID4	hsa-miR-374b-5p, hsa-miR-374a-5p	2.88E-11	0.015565322
MAGI2-AS3	PARD6B	hsa-miR-374b-5p, hsa-miR-374a-5p	0.013783334	0.033354261
MAGI2-AS3	MEIS1	hsa-miR-374b-5p, hsa-miR-374a-5p	2.38E-34	0.004970439
MAGI2-AS3	TTLL7	hsa-miR-374b-5p, hsa-miR-374a-5p	0.006401492	0.003557788
MAGI2-AS3	DST	hsa-miR-374b-5p, hsa-miR-374a-5p	1.62E-19	0.000732486
MAGI2-AS3	LRCH2	hsa-miR-374b-5p, hsa-miR-374a-5p	9.57E-26	0.010621043
MAGI2-AS3	NOVA1	hsa-miR-374b-5p, hsa-miR-374a-5p	0.036152925	0.003557788
SNHG3	PITX2	hsa-miR-340-5p	1.08E-06	0.028880866
SNHG3	GPT2	hsa-miR-340-5p	2.34E-06	0.036101083
SNHG3	RCC1	hsa-miR-340-5p	5.44E-09	0.010830325
SNHG3	PLK4	hsa-miR-340-5p	0.013853232	0.003610108
SNHG3	PUS7	hsa-miR-340-5p	2.11E-06	0.028880866
SNHG3	DBF4	hsa-miR-340-5p	0.003173507	0.02166065
SNHG3	H1F0	hsa-miR-340-5p	0.026006197	0.010830325
SNHG3	SLFN13	hsa-miR-340-5p	0.007852904	0.010830325
SNHG3	SKP2	hsa-miR-340-5p	0.00072561	0.003610108
AC074117.1	E2F2	hsa-let-7a-5p, hsa-let-7i-5p, hsa-let-7c-5p, hsa-let-7b-5p, hsa-let-7d-5p, hsa-miR-98-5p	1.10E-16	0.000223994
FBXL19-AS1	PDIA4	hsa-miR-422a, hsa-miR-378a-3p	0.00076262	0.005460401
FBXL19-AS1	CLK2	hsa-miR-422a, hsa-miR-378a-3p	4.13E-17	0.044388607
FBXL19-AS1	PLEKHG2	hsa-miR-422a, hsa-miR-378a-3p	1.73E-14	0.000941767
H19	UBE2C	hsa-miR-138-5p	7.38E-06	0.0433213
H19	DEPDC1	hsa-miR-130a-3p, hsa-miR-454-3p, hsa-miR-130b-3p	0.000140905	0.011023334
H19	CEP55	hsa-miR-130a-3p, hsa-miR-454-3p, hsa-miR-130b-3p	0.000210262	0.017341521
H19	GPT2	hsa-miR-130a-3p, hsa-miR-454-3p, hsa-miR-130b-3p	1.09E-05	0.006323082
H19	MYBL2	hsa-miR-29c-3p, hsa-miR-29b-3p, hsa-miR-29a-3p	8.96E-08	0.00310273
H19	SASH1	hsa-miR-4295, hsa-miR-130a-3p, hsa-miR-454-3p, hsa-miR-130b-3p, hsa-miR-3666	9.52E-07	0.002989001
H19	PEAK1	hsa-miR-29c-3p, hsa-miR-29b-3p, hsa-miR-29a-3p	0.024013934	0.021136246
H19	NDST1	hsa-miR-29c-3p, hsa-miR-29b-3p, hsa-miR-29a-3p	2.87E-08	0.004538761
H19	CCNA2	hsa-miR-130a-3p, hsa-miR-454-3p, hsa-miR-130b-3p	0.001073861	0.046656577
H19	CCNE1	hsa-miR-107, hsa-miR-138-5p, hsa-miR-103a-3p	0.015151623	0.021136246
H19	HEG1	hsa-miR-4295, hsa-miR-130a-3p, hsa-miR-454-3p, hsa-miR-130b-3p, hsa-miR-3666	5.35E-05	0.004199543
H19	SEMA6A	hsa-miR-107, hsa-miR-138-5p, hsa-miR-103a-3p	3.15E-05	0.01397365
H19	ESCO2	hsa-miR-130a-3p, hsa-miR-454-3p, hsa-miR-130b-3p	0.004980968	0.001165048
H19	FAM136A	hsa-miR-29c-3p, hsa-miR-29b-3p, hsa-miR-29a-3p	5.91E-05	0.006323082
H19	TRAF4	hsa-miR-29c-3p, hsa-miR-29b-3p, hsa-miR-29a-3p	0.042237801	0.000597274
H19	E2F2	hsa-miR-4295, hsa-miR-130a-3p, hsa-miR-454-3p, hsa-miR-130b-3p, hsa-miR-3666	1.05E-07	0.024521918
H19	SOX4	hsa-miR-4295,hsa-miR-130a-3p,hsa-miR-138-5p,hsa-miR-454-3p,hsa-miR-130b-3p, hsa-miR-3666	4.19E-07	0.037463
H19	CPS1	hsa-miR-29c-3p, hsa-miR-29b-3p, hsa-miR-29a-3p	6.30E-06	0.000597274
H19	COL1A1	hsa-miR-29c-3p, hsa-miR-29b-3p, hsa-miR-107, hsa-miR-103a-3p, hsa-miR-29a-3p	5.76E-06	0.004199543
H19	TSPAN18	hsa-miR-130a-3p, hsa-miR-454-3p, hsa-miR-130b-3p	0.002343508	0.011023334
H19	PDK1	hsa-miR-130a-3p, hsa-miR-138-5p, hsa-miR-454-3p, hsa-miR-130b-3p	0.000948766	0.004507133
H19	RAPGEF4	hsa-miR-4295, hsa-miR-130a-3p, hsa-miR-454-3p, hsa-miR-130b-3p, hsa-miR-3666	0.035046485	0.004199543
H19	E2F7	hsa-miR-29c-3p, hsa-miR-29b-3p, hsa-miR-4295, hsa-miR-130a-3p, hsa-miR-454-3p, hsa-miR-130b-3p, hsa-miR-3666, hsa-miR-29a-3p	1.92E-07	0.001396262
H19	EGLN3	hsa-miR-130a-3p, hsa-miR-454-3p, hsa-miR-130b-3p	2.18E-10	0.025364366
H19	SIX4	hsa-miR-107, hsa-miR-4295, hsa-miR-130a-3p, hsa-miR-454-3p, hsa-miR-103a-3p, hsa-miR-130b-3p	0.000445785	0.023400997
H19	RNF122	hsa-miR-29c-3p, hsa-miR-29b-3p, hsa-miR-29a-3p	0.000761801	0.006323082
H19	ZFYVE9	hsa-miR-4295, hsa-miR-130a-3p, hsa-miR-454-3p, hsa-miR-130b-3p, hsa-miR-3666	4.46E-05	0.000516124
H19	KDM5B	hsa-miR-29c-3p, hsa-miR-29b-3p, hsa-miR-29a-3p	0.001550086	0.025364366
H19	NHSL1	hsa-miR-4295, hsa-miR-130a-3p, hsa-miR-454-3p, hsa-miR-130b-3p	0.000412253	0.002162624
H19	COL7A1	hsa-miR-29c-3p, hsa-miR-29b-3p, hsa-miR-29a-3p	1.85E-09	0.001165048
H19	CCDC137	hsa-miR-130a-3p, hsa-miR-454-3p, hsa-miR-130b-3p	1.74E-05	0.021136246
H19	COL5A2	hsa-miR-29c-3p, hsa-miR-29b-3p, hsa-miR-29a-3p	4.30E-07	0.000244951
H19	TTYH3	hsa-miR-4295, hsa-miR-130a-3p, hsa-miR-454-3p, hsa-miR-130b-3p, hsa-miR-3666	1.46E-08	0.000863481
H19	RFLNB	hsa-miR-29c-3p, hsa-miR-29b-3p, hsa-miR-29a-3p	2.44E-07	0.004538761
H19	FIBIN	hsa-miR-130a-3p, hsa-miR-454-3p, hsa-miR-130b-3p	0.023475262	0.001988426
H19	LRCH1	hsa-miR-4295, hsa-miR-130a-3p, hsa-miR-454-3p, hsa-miR-130b-3p, hsa-miR-3666	0.00742055	0.020020653
H19	FZD4	hsa-miR-130a-3p, hsa-miR-454-3p, hsa-miR-130b-3p	0.000109712	0.025364366
H19	SOX12	hsa-miR-29c-3p, hsa-miR-29b-3p, hsa-miR-370-3p, hsa-miR-29a-3p	2.77E-06	0.000843206
H19	SMOC1	hsa-miR-4295, hsa-miR-130a-3p, hsa-miR-454-3p, hsa-miR-130b-3p, hsa-miR-3666	9.41E-06	0.001685168
H19	SPOCK1	hsa-miR-130a-3p, hsa-miR-454-3p, hsa-miR-130b-3p	7.87E-14	0.040677495
H19	GPR37	hsa-miR-29c-3p, hsa-miR-29b-3p, hsa-miR-29a-3p	0.00643677	0.000597274
H19	HOXA5	hsa-miR-4295, hsa-miR-130a-3p, hsa-miR-454-3p, hsa-miR-130b-3p, hsa-miR-3666	0.001830419	0.006644408
H19	SLC16A14	hsa-miR-29c-3p, hsa-miR-29b-3p, hsa-miR-29a-3p	3.04E-06	0.011023334
H19	RACGAP1	hsa-miR-130a-3p, hsa-miR-454-3p, hsa-miR-130b-3p	0.000189766	0.025364366
H19	PTGFRN	hsa-miR-4295, hsa-miR-130a-3p, hsa-miR-454-3p, hsa-miR-130b-3p, hsa-miR-3666	1.28E-07	0.01439497
H19	TCF4	hsa-miR-29c-3p, hsa-miR-29b-3p, hsa-miR-4295, hsa-miR-130a-3p, hsa-miR-138-5p, hsa-miR-454-3p, hsa-miR-130b-3p, hsa-miR-3666, hsa-miR-29a-3p	0.011403769	0.000309855
H19	PTHLH	hsa-miR-29c-3p, hsa-miR-29b-3p, hsa-miR-107, hsa-miR-103a-3p, hsa-miR-29a-3p	5.53E-10	0.0035563
H19	DIO2	hsa-miR-29c-3p, hsa-miR-29b-3p, hsa-miR-29a-3p	0.000723056	0.004538761
H19	CCT6A	hsa-miR-130a-3p, hsa-miR-454-3p, hsa-miR-130b-3p	1.84E-05	0.017341521
H19	OTUB2	hsa-miR-29c-3p, hsa-miR-29b-3p, hsa-miR-29a-3p	0.030508582	0.001988426
H19	NPTX1	hsa-miR-4295, hsa-miR-130a-3p, hsa-miR-454-3p, hsa-miR-130b-3p, hsa-miR-3666	0.001417033	0.01439497
H19	CDC7	hsa-miR-29c-3p, hsa-miR-29b-3p, hsa-miR-29a-3p	6.28E-05	0.008478302
H19	LOXL2	hsa-miR-29c-3p, hsa-miR-29b-3p, hsa-miR-29a-3p	1.52E-09	0.001988426
H19	ANKRD13B	hsa-miR-29c-3p, hsa-miR-29b-3p, hsa-miR-138-5p, hsa-miR-29a-3p	1.56E-08	0.002814389
H19	AKT3	hsa-miR-29c-3p, hsa-miR-29b-3p, hsa-miR-107, hsa-miR-103a-3p, hsa-miR-29a-3p	7.18E-14	0.011331535
H19	MLLT11	hsa-miR-29c-3p, hsa-miR-29b-3p, hsa-miR-29a-3p	2.39E-13	0.001165048
H19	HMGCS1	hsa-miR-29c-3p, hsa-miR-29b-3p, hsa-miR-29a-3p	0.000773742	0.01397365
H19	TUBB2A	hsa-miR-29c-3p, hsa-miR-29b-3p, hsa-miR-29a-3p	0.014254037	0.000244951
H19	KIF26A	hsa-miR-29c-3p, hsa-miR-29b-3p, hsa-miR-29a-3p	0.001301997	0.001988426
H19	KIAA1211	hsa-miR-4295, hsa-miR-130a-3p, hsa-miR-454-3p, hsa-miR-130b-3p, hsa-miR-3666	8.34E-06	0.002491825
H19	PI15	hsa-miR-29c-3p, hsa-miR-29b-3p, hsa-miR-29a-3p	0.000177879	0.001988426
H19	HOXB3	hsa-miR-4295, hsa-miR-130a-3p, hsa-miR-454-3p, hsa-miR-130b-3p, hsa-miR-3666	4.10E-05	0.004924644
H19	MAP 2 K6	hsa-miR-29c-3p,hsa-miR-29b-3p,hsa-miR-29a-3p	0.002517394	0.00310273
H19	PRKAA2	hsa-miR-130a-3p, hsa-miR-454-3p, hsa-miR-130b-3p	0.000960667	0.035134428
H19	COL4A5	hsa-miR-29c-3p, hsa-miR-29b-3p, hsa-miR-29a-3p	0.010618506	0.046656577
H19	SLC16A1	hsa-miR-29c-3p, hsa-miR-29b-3p, hsa-miR-29a-3p	6.26E-06	0.011023334

### Risk assessment analysis of the prognostic value of key RNAs in ceRNA networks

**Fig. 3:**
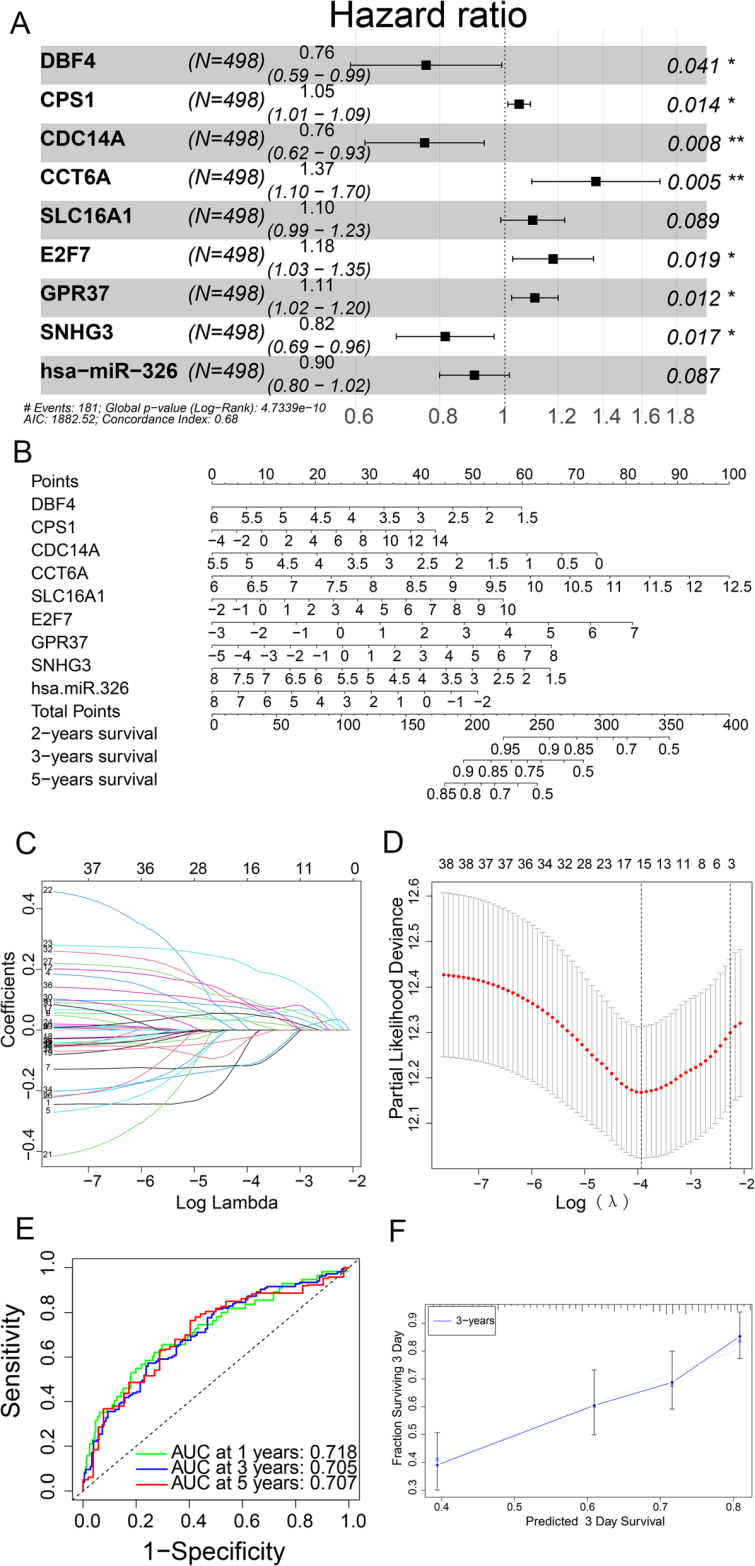
Multivariate Cox analysis and gene model diagnostic process of LUAD Regressions of multivariate Cox (A), the nomogram plots of clinical variables in TCGA LUAD patients (B), the model diagnostic process of Lasso regression (C-D), ROC curve assessed the sensitivity and specificity of nine key genes of ceRNA network as diagnostic biomarkers for LUAD in TCGA, (E) and the nomogram calibration curves of 3-year survival (F).

In this study, 38 genes in the ceRNA network were analysed utilizing univariate Cox regression, and nine genes were screened and tested utilizing multivariate Cox regression. Nine genes (DBF4, CPS1, CDC14A, CCT6A, SLC16A1, E2F7, GPR37, SNHG3, Hsa-miR-326) in the ceRNA network were merged into the Cox proportional-risk model to assess its prognostic value (Fig. 3A). A nomogram was established using the risk score of the prediction model and clinical variables to calculate the overall survival rate of LUAD patients at the first, third, and fifth years (Fig. 3B). After adopting multivariate cox analysis, Lasso regression was perfromed to assess the stability of the genetic model (Fig. 3C and D). The time-dependent ROC curves and calibration curves (Fig. 3E and F) showed good accuracy for the area under the curve (AUC) as well as the calibration of the nomogram. The AUC values for first-year, third-year, and fifth-year survivalbility were 0.718, 0.705, and 0.707, respectively.

### Preliminary verification of clinical specimens

**Fig. 4:**
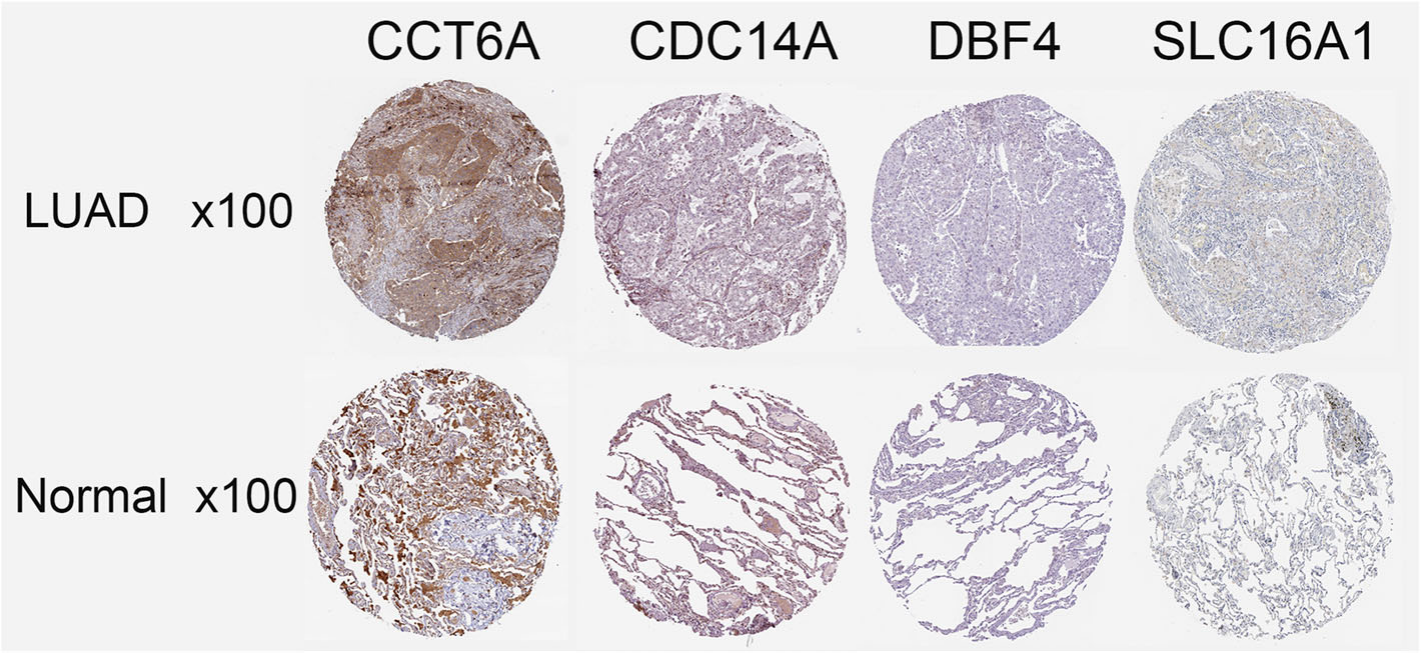
Results of preliminary clinical specimen validation Immunohistochemical findings suggested that CCT6A, CDC14A, DBF4, and SLC16A1 proteins were significantly different in diagnosing normal tissues and LUAD.

To verify the expression of key genes in tissues, we searched through The Human Protein Atlas database (https://www.proteinatlas.org/). Immunohistochemical results showed that CCT6A, CDC14A, DBF4, and SLC16A1 proteins were showing different attributes in differentiating healthy tissues and LUAD (Fig. 4).

### Risk-scoring models as independent predictors for LUAD prognosis

**Fig. 5:**
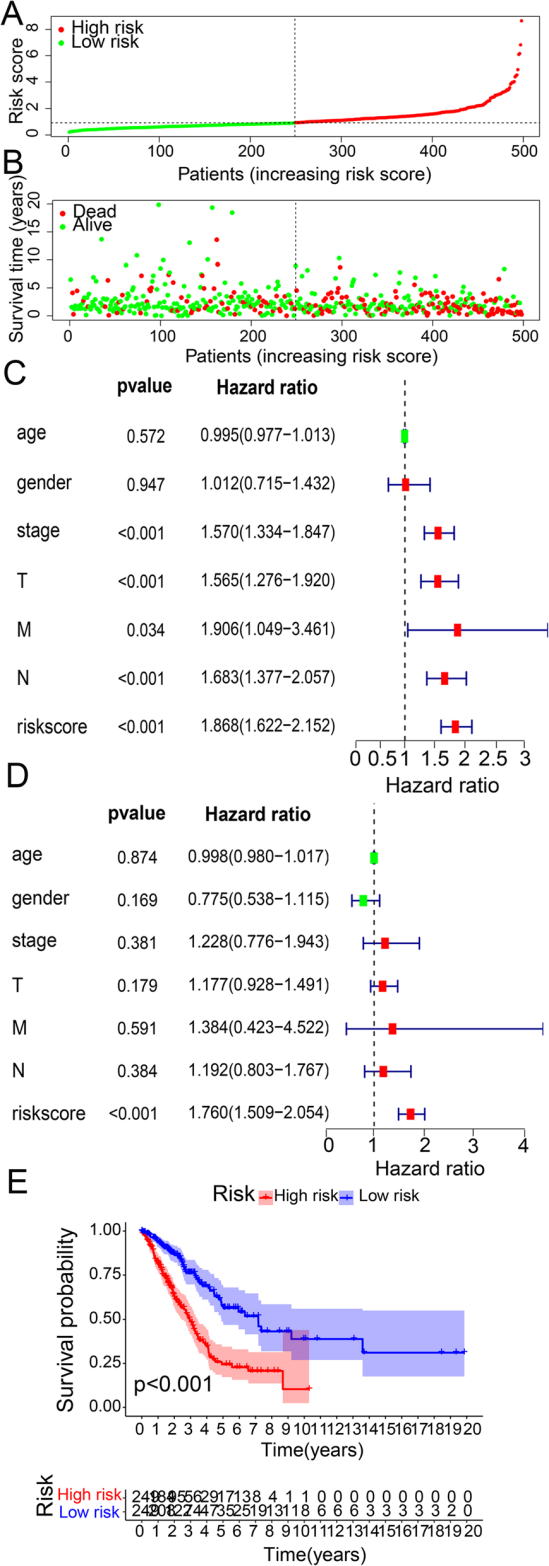
Risk score model as an independent predictor of prognosis in LUAD (A) the scatter plot of risk scores for LUAD patients, (B) the scatter plot of LUAD patients’ survival time, (C) the univariate cox regression between risk scores and clinical parameters, (D) the multivariate cox regression between risk scores and clinical parameters, and (E) the Kaplan-Meier survival curves for the high- and low-risk groups.

The risk scores and survivalbility of LUAD patients are shown in Fig. 5A and B, respectively, and LUAD patients were separated into high and low risk groups in terms of risk scores. To investigate whether the constructed risk score model was independent of clinicopathological parameters, the univariate and multivariate Cox regression analysis for age, sex, stage, TNM, and risk score were performed. In univariate Cox model, pathologic T, N stage, pathologic stage, and high-risk score were related to poor survivalbility (Fig. 5C). And Fig. 5D shows that in multivariate Cox model, the risk score was the sole parameter that could independently predict the overall survivalbility. The Kaplan-Meier survival curve was constructed for the high and low risk groups with a statistically meaningful difference in the survivalbility between the two groups (*p* < 0.001), and The low-risk group had a superior OS than that the high-risk group (Fig. 5E).

Based on the above findings, we further designed a validation cohort to confirm the potential value of risk-score model.

levels in predicting LUAD prognosis. Twenty patients with LUAD were enrolled. LUAD patients were divided into two high- and low-risk groups based on the levels of risk score (median value), patient survival of low- and high-risk score groups was analyzed by Fisher’s exact test at 1 year of return visit. As shown in the contingency table, high risk score group was correlated with poor outcome of LUAD patients (Table 3), which suggested the expression level of nine genes have a high prognostic value in patients with LUAD.

**Table 3:** Contingency table of high-low risk score groups for predicting survival in clinical LUAD samples

Survival	Death	Alive	Total number of rows
Risk score			
High	4	6	10
Low	1	9	10
Total number of columns	5	15	20

### The composition of TIICs between the LUAD high- and low-risk scores

**Fig. 6:**
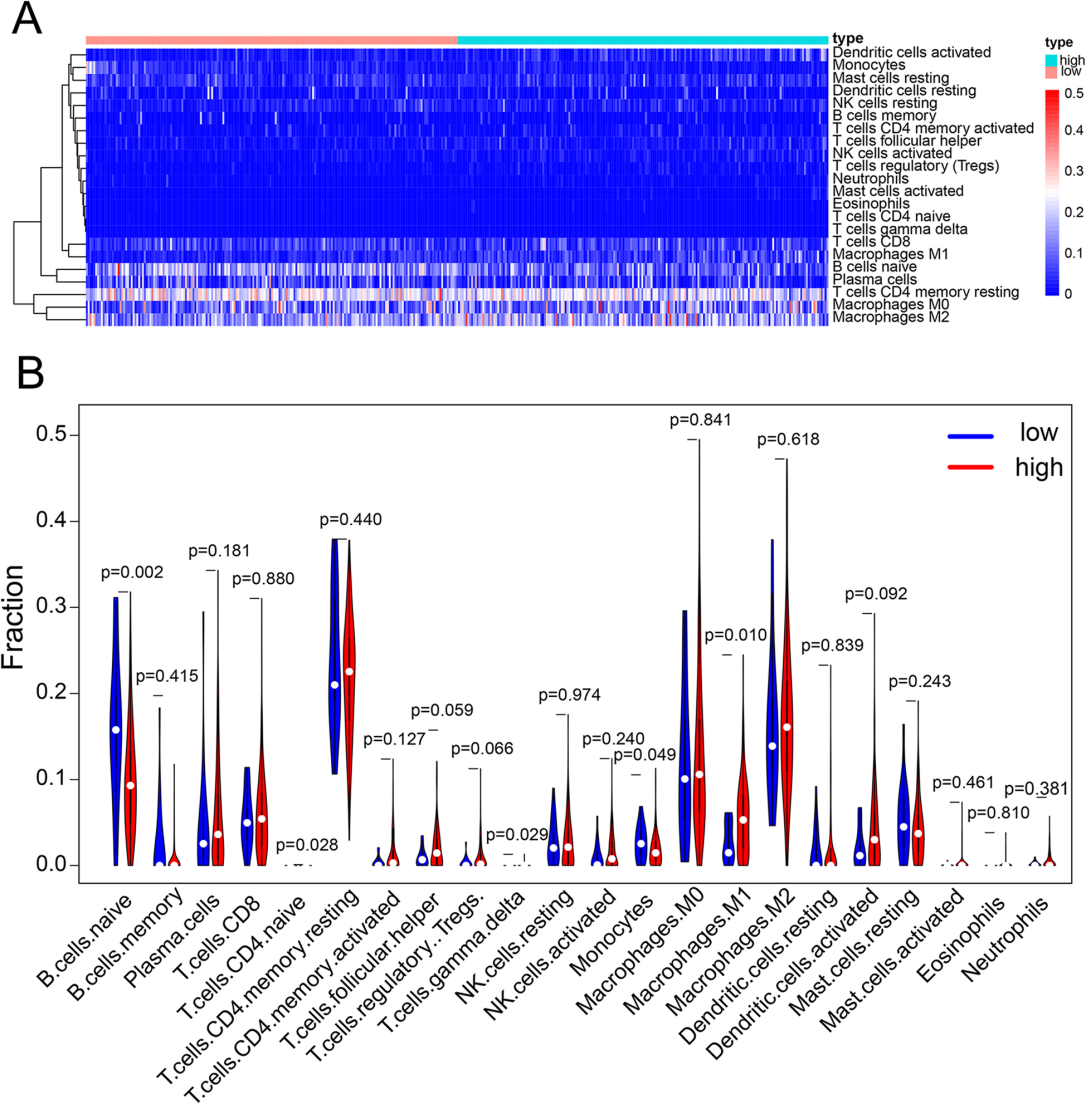
The composition of TIICs in LUAD tissues for the high- and low-risk groups (A) significant tumor-infiltrating immune cells in LUAD of high- and low-risk groups, (B) CIBERSORT identified cell types by determining the relative subset of RNA transcripts.

As illustrated in Fig. 6, the histogram (Fig. 6A) and heat maps (Fig. 6B) were generated from the CIBERSORT algorithm for assessing the composition of significant TIICs in LUAD tissues of high and low risk groups. In both groups, the T-cell CD4, T-cell CD8, macrophage M0, and macrophage M2 were significantly demonstrated in LUAD tissues. The Wilcoxon rank-sum test showed that five immune cell components, i.e., B-cell naïve (*p* = 0.002), T-cell CD4 naïve (*p* = 0.028), T-cell gamma δ (*p* = 0.029), monocytes (*p* = 0.049), and macrophage M1 (*p* = 0.010), were significantly different in LUAD tissues between the high and low risk groups (Fig. 6B).

### The co-expression of genes and immune cells for prognosis

**Fig. 7:**
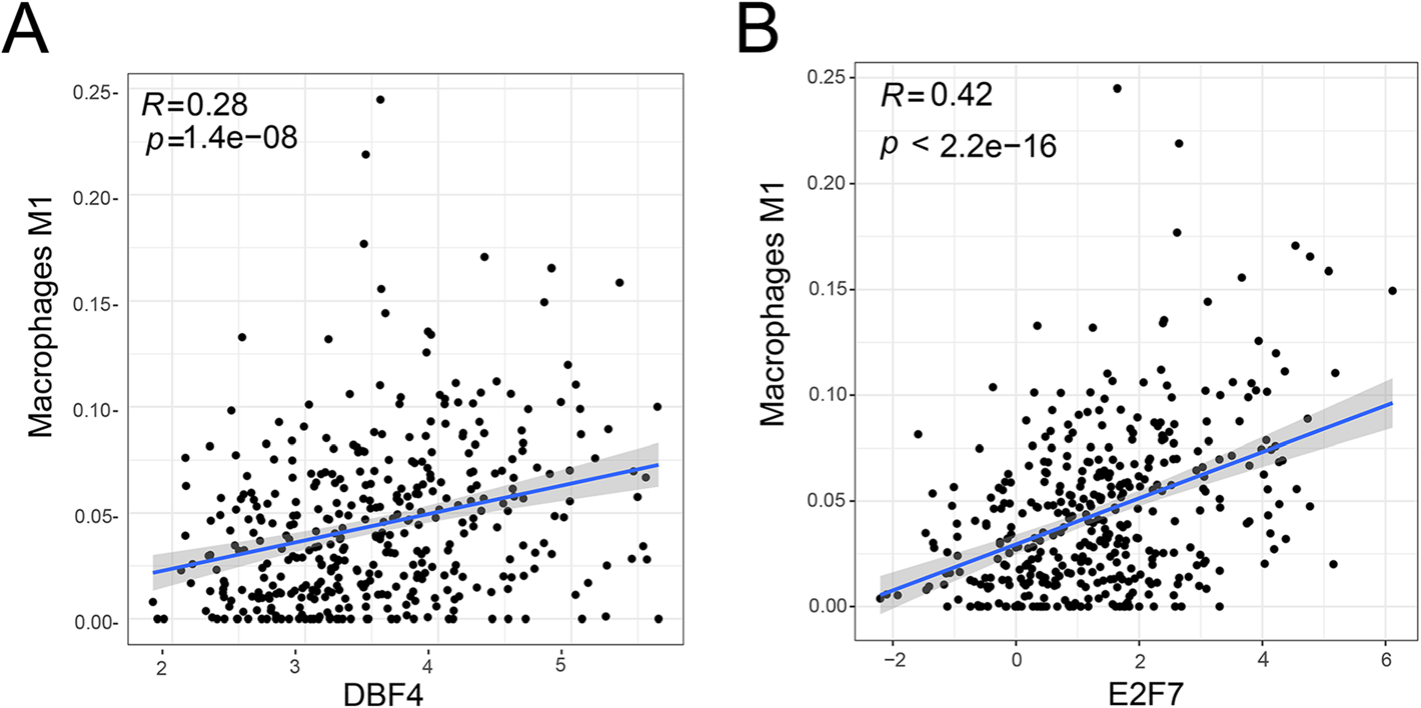
Co-expression of TIICs and principal members of the ceRNA network E2F7 and macrophage M1 (*R* = 0.42, *p* < 2.2e^− 16^), (B) DBF4 and macrophage M1 (*R* = 0.28, *p* = 1.4e^− 08^).

In order to explore the role of key genes in the ceRNA network and tumor immune cells played during prognosis of LUAD, co-expression analysis of key genes in the ceRNA network and different TIICs acquired from LUAD samples from high and low risk groups were performed. The result shows that the E2F7 gene and macrophage M1 (the correlation coefficient, *R* = 0.42, *p* < 2.2e ^− 16^), DBF4 and macrophage M1 (*R* = 0.28, *p* = 1.4e^− 08^) displayed a positive correlation (Fig. 7).

## Discussion

In this study, a bioinformatics approach was used to predict lung cancer progression and metastasis based on RNA sequencing data extracted from the TCGA database, and 93 DE mRNAs, seven DE lncRNAs, and 15 DE miRNAs were found to be dysregulated in LUAD tissues. The relationship between differentially expressed RNAs in the ceRNA network and the clinical prognosis of LUAD patients showed that key genes could well-predict LUAD. Besides, the Cox proportional-risk model might serve as an unconstrained factor in predicting LUAD. Looking at the nomogram of the nine key genes (hsa-miR-326, DBF4, CPS1, CDC14A, CCT6A, SLC16A1, E2F7, GPR37, SNHG3), the AUC values could mostly predict LUAD clinically. Also, four highly expressed LUAD-associated immune infiltrating cells (macrophage M0, macrophage M2, T-cell CD4 memory dormancy, and B-cell naïve) related to LUAD and cancer paracellular were identified via the CIBERSORT algorithm, and five immune cells (B-cell naïve, T-cell CD4 naïve, T-cell gamma delta, monocytes, macrophage M1) showed significantly difference in high and low risk LUAD groups. The most significant difference in the high- and low-risk group was B cells (*P* = 0.002), which are important members of the body involved in humoral immunity, so the decrease in B cells in the high-risk group may be one of the reasons for the poor survival. Additionally, two pairs of important biomarkers related to the growth of LUAD were identified, and initial clinical specimen validation showed that E2F7, macrophage M1 (*R* = 0.42, *p* < 2.2e^− 16^), and DBF4 and macrophage M1 (*R* = 0.28, *p* = 1.4e^− 08^) were significantly associated with the attributes of co-expression. And their associations are crucial in predicting LUAD.

In recent years, miRNA is one of the hot topics in tumor research, largely because aberrant expression of miRNA is related to tumorigenesis and progression of multiple tumors [15]. This study predicted that 15 DE miRNAs were linked to the development of LUAD. In particular, aberrant expression of hsa-miR-326 (miR-326) is involved in a variety of pathological processes, including endometrial cancer, gastric cancer, lung cancer, osteosarcoma, pulmonary fibrosis, and breast cancer. Thus, it is used as a biomarker for identifying cancer, treatment, and prognosis [16–21]. As a repressor of the Hedgehog signaling pathway, miR-326 controls the growth of cerebellar neuronal progenitors and cancer cells [22]. It is also found on patients with type I diabetics and leukemia [23–25]. Meanwhile, low miR-326 expression in gastric tumor is related to clinical stage, tumor depth, lymph node metastasis, and distant metastasis; it is a relatively poorly constrained prognostic factor for gastric tumor [26]. In glioblastoma tissues, miR-326 was downregulated and involved in tumorigenesis and progression of glioma, and small amount of miR-326 expression was correlated with clinicopathological factors and prognosis of glioma [27]. However, studies on hsa-miR-326 and the development of LUAD are generally scarce.

Immune infiltration of the tumor microenvironment is an important factor affecting the immune response and prognosis. Macrophages, which are crucial in the metastatic process, are major components of TIICs and often trigger local inflammation [28]. In this study, macrophages in tumor bodies were divided into M1 and M2 types. In the early stages of tumor development, macrophages either act as phagocytose individual tumor cells or act as antigen presenting cells (APCs) to trigger an immune response of CD8+ T cells. Eventually, when CD8+ T cells are unable to generate enough immune effect, tumor-associated macrophages (TAMs) would secretly stimulate tumor growth or angiogenesis [29]. However, macrophage infiltration in the tumor stroma is a negative prognostic factor for LUAD [30]. In NSCLC, TAMs would stimulate tumor metastasis via the TGF- inhibitor/SOX9 axis [31]. Specifically, the M2 subtype stimulates the invasion of lung cancer cells, while the M1 subtype suppresses tumor formations [32]. As a result, macrophages are a key factor in the metastatic process of LUAD [33].

E2F7 is a member of the family of E2F transcription factors (E2Fs), which plays an important role in cell proliferation, differentiation, and apoptosis. There are eight genes in the E2F family, designated as E2F1 - E2F8 in the order of discovery. E2F7 abnormalities seem to have a crucial function in the growth of cancer cells. In breast-cancer patients treated with tamoxifen, the abundant apperence of E2F7 is associated with a high possibility of recurrence and poor prognosis. E2F7 usually competes with E2F1 to suppress miR-15a/16 clustering [34]. The acquisition of the E2F7 function counteracted the effects of miR-30a-5p on cell propagation and metastasis [35]. The abundant aperence of E2F7 shown within NSCLC tissues usually correlated with poor prognosis. It usually inhibits cell propagation, migration, invasion, development of cancer, EMT and AKT pathways in NSCLC cells by targeting miR-935 [36]. However, related studies are scarce.

In summary, this study analysed DE mRNAs, lncRNAs, and miRNAs in LUAD cancer and para cancer using an integrative biological approach. A ceRNA network of lncRNA-miRNA-mRNA ceRNA was constructed, uncovering a potentially new regulatory mechanism. The relationship between DE mRNAs, lncRNAs, and miRNAs in the ceRNA network and a Cox proportional-risk model in ceRNAs was examined to predict LUAD prognosis. This risk-assessment model could serve as an independent factor to predict LUAD prognosis. In LUAD tissues, five immune cell types with significant differences were identified using the CIBERSORT algorithm and co-expression analysis, revealing significant correlations between E2F7 and macrophage M1 (*R* = 0.42, *p* < 2.2e^− 16^) and DBF4 and macrophage M1 (*R* = 0.28, *p* = 1.4e^− 08^). These two pairs of co-expressed genes and their associated mechanisms would play an important role in predicting LUAD prognosis.

## Supplementary Information

**Additional file 1: Supplementary Table 1** Thirty-eight genes significantly associated with prognosis in LUAD

**Additional file 2: Supplementary Table 2** Risk assessment of 20 clinical LUAD patients

## Data Availability

The following information was supplied regarding data availability: All the original data in this study were downloaded from the public databases including TCGA (https://portal.gdc.cancer.gov/).
