# Supplementary material for: The construction and analysis of ceRNA network and patterns of immune infiltration in lung adenocarcinoma
Source: BMC Cancer. 2021 Nov 16;21:1228. doi: 10.1186/s12885-021-08932-z (PMC8594182; doi:10.1186/s12885-021-08932-z)
Supplement: Supplementary file 1 — Additional file 1: Supplementary Table 1 Thirty-eight genes significantly associated with prognosis in LUAD [file 12885_2021_8932_MOESM1_ESM.docx]

Supplementary table 1

| ID | HR | HR.95L | HR.95H | P value |
| --- | --- | --- | --- | --- |
| DBF4 | 1.222446573 | 1.024725582 | 1.458317866 | 0.025661195 |
| E2F2 | 1.137817894 | 1.004309997 | 1.289073655 | 0.042611295 |
| CPS1 | 1.055061409 | 1.01777222 | 1.093716802 | 0.003505879 |
| DEPDC1 | 1.229430503 | 1.112796 | 1.358289716 | 4.88E-05 |
| FAM136A | 1.333398927 | 1.025538147 | 1.733677781 | 0.031694592 |
| ADRB1 | 0.914122489 | 0.856095522 | 0.976082579 | 0.007287061 |
| CDC14A | 0.826168252 | 0.688666528 | 0.991124082 | 0.039786952 |
| PTHLH | 1.074983056 | 1.000758143 | 1.154713134 | 0.047622151 |
| CDC7 | 1.193835555 | 1.035828812 | 1.375944862 | 0.014447301 |
| MYBL2 | 1.148662353 | 1.054467393 | 1.251271693 | 0.001499151 |
| CCNE1 | 1.180007218 | 1.067777825 | 1.304032545 | 0.00117013 |
| GPI | 1.504092259 | 1.223743236 | 1.848666825 | 0.000105106 |
| COL1A1 | 1.113008591 | 1.016299799 | 1.218919972 | 0.020966405 |
| COL7A1 | 1.116214015 | 1.036110029 | 1.202511018 | 0.003808647 |
| RALGPS2 | 1.249931098 | 1.047722107 | 1.491166159 | 0.013222081 |
| EGLN3 | 1.140777476 | 1.052831648 | 1.236069654 | 0.001292145 |
| LOXL2 | 1.259823092 | 1.135404775 | 1.39787524 | 1.34E-05 |
| PTGFRN | 1.305088156 | 1.079485968 | 1.577839033 | 0.005962372 |
| TTYH3 | 1.195453763 | 1.004583891 | 1.422588708 | 0.044273326 |
| CEP55 | 1.247272301 | 1.115806506 | 1.394227568 | 0.000100998 |
| PLK4 | 1.272121893 | 1.110685664 | 1.457022598 | 0.000508773 |
| CCNA2 | 1.291063032 | 1.154280252 | 1.444054639 | 7.78E-06 |
| CCT6A | 1.528647736 | 1.287464944 | 1.815011672 | 1.27E-06 |
| SPOCK1 | 1.125599578 | 1.053846267 | 1.202238362 | 0.000430684 |
| SLC16A1 | 1.201769572 | 1.099549208 | 1.313492925 | 5.07E-05 |
| RACGAP1 | 1.296504069 | 1.126482568 | 1.49218714 | 0.000293972 |
| E2F7 | 1.256626565 | 1.136909681 | 1.388949668 | 7.75E-06 |
| GPR37 | 1.152831798 | 1.067309783 | 1.245206569 | 0.000298784 |
| ESCO2 | 1.233555653 | 1.091507157 | 1.394090309 | 0.000771824 |
| UBE2C | 1.175648695 | 1.073814205 | 1.287140595 | 0.000464282 |
| LMNB2 | 1.386798094 | 1.167259688 | 1.647627322 | 0.000200124 |
| CCDC137 | 1.540846263 | 1.193172808 | 1.98982678 | 0.00092084 |
| COL5A2 | 1.14906395 | 1.033555054 | 1.277481985 | 0.010153749 |
| SNHG3 | 0.841278886 | 0.718504351 | 0.985032538 | 0.031766928 |
| hsa-let-7c-5p | 0.850117152 | 0.741550348 | 0.974578696 | 0.019840737 |
| hsa-miR-21-5p | 1.25311087 | 1.018980316 | 1.541037475 | 0.032505171 |
| hsa-miR-29b-3p | 0.863279506 | 0.745421003 | 0.999772614 | 0.049646124 |
| hsa-miR-326 | 0.887380339 | 0.791308144 | 0.995116595 | 0.040983408 |
