# Supplementary material for: The construction and analysis of ceRNA network and patterns of immune infiltration in lung adenocarcinoma
Source: BMC Cancer. 2021 Nov 16;21:1228. doi: 10.1186/s12885-021-08932-z (PMC8594182; doi:10.1186/s12885-021-08932-z)
Supplement: Supplementary file 2 — Additional file 2: Supplementary Table 2 Risk assessment of 20 clinical LUAD patients [file 12885_2021_8932_MOESM2_ESM.docx]

Supplementary table 2

| id | Fustate | DBF4 | CPS1 | CDC14A | CCT6A | SLC16A1 | E2F7 | GPR37 | SNHG3 | hsa-miR-326 | riskscore | risk |
| --- | --- | --- | --- | --- | --- | --- | --- | --- | --- | --- | --- | --- |
| 1 | 0 | 2.7200 | 0.2328 | 0.0634 | 5.0703 | 1.9940 | 3.6810 | 3.1010 | 9.6405 | 7.1759 | -0.7634 | high |
| 2 | 0 | 3.1991 | 0.7413 | 0.0895 | 4.0541 | 1.7034 | 3.7309 | 3.1866 | 8.3830 | 9.9005 | -1.2285 | low |
| 3 | 0 | 1.9453 | 1.0116 | 0.0621 | 3.6755 | 1.8549 | 3.7626 | 2.9748 | 9.8700 | 10.3602 | -1.3401 | low |
| 4 | 0 | 3.3830 | 0.7346 | 0.1732 | 4.0233 | 2.0101 | 4.0377 | 4.0784 | 9.6426 | 8.1995 | -1.2205 | low |
| 5 | 1 | 2.1074 | 1.0447 | 0.2696 | 6.8555 | 1.7921 | 3.8359 | 2.9657 | 9.6979 | 7.4805 | -0.1106 | high |
| 6 | 0 | 3.0941 | 0.3929 | 0.0426 | 3.6476 | 2.2457 | 3.7892 | 2.9493 | 11.0847 | 6.9952 | -1.5429 | low |
| 7 | 0 | 3.2462 | 0.5864 | 0.0029 | 4.1868 | 1.8984 | 3.8141 | 2.8944 | 10.4030 | 7.2949 | -1.3225 | low |
| 8 | 1 | 3.1984 | 0.1557 | 0.0841 | 4.3778 | 2.0314 | 3.8628 | 3.0648 | 8.5191 | 7.6430 | -0.9074 | low |
| 9 | 0 | 2.6702 | 0.6241 | 0.2472 | 5.0592 | 1.7728 | 4.5470 | 3.1313 | 9.9416 | 7.7251 | -0.7784 | high |
| 10 | 0 | 3.3038 | 0.4901 | 0.2055 | 3.9607 | 1.7986 | 3.7717 | 3.3008 | 9.8813 | 9.2479 | -1.5404 | low |
| 11 | 0 | 3.4284 | 1.1361 | 0.1203 | 5.7988 | 2.1062 | 4.9725 | 2.9649 | 10.8892 | 8.0512 | -0.8354 | high |
| 12 | 0 | 2.0425 | 0.7128 | 0.1336 | 3.5880 | 2.0458 | 4.0048 | 2.9287 | 7.0828 | 8.0793 | -0.5687 | high |
| 13 | 1 | 3.4060 | 1.1894 | 0.0115 | 5.9819 | 1.9331 | 5.2374 | 3.5443 | 7.8323 | 7.9122 | -0.0155 | high |
| 14 | 0 | 2.7306 | 0.5877 | 0.0365 | 4.0041 | 1.8731 | 3.7907 | 3.0081 | 10.8517 | 10.3087 | -1.6488 | low |
| 15 | 0 | 1.8785 | 0.1993 | 0.4626 | 4.8918 | 2.3508 | 3.8394 | 3.0381 | 8.1556 | 9.6118 | -0.6000 | high |
| 16 | 0 | 3.3886 | 1.2042 | 0.2750 | 3.7095 | 1.8000 | 3.8767 | 3.0782 | 7.9972 | 6.9294 | -1.0053 | low |
| 17 | 1 | 1.9563 | 0.2881 | 0.5617 | 5.0232 | 2.0637 | 3.8637 | 3.4988 | 7.3016 | 7.4837 | -0.1837 | high |
| 18 | 1 | 1.8415 | 0.9406 | 0.6368 | 7.3506 | 1.8549 | 3.7862 | 3.2367 | 8.7247 | 7.1524 | 0.2672 | high |
| 19 | 0 | 3.5951 | 0.4055 | 0.5811 | 3.9240 | 1.9125 | 3.8210 | 3.0645 | 7.2179 | 8.3597 | -1.1074 | low |
| 20 | 0 | 1.8008 | 0.4860 | 0.1801 | 3.9266 | 2.1060 | 3.8928 | 3.1217 | 9.5326 | 7.1617 | -0.8195 | high |
